# Development of Green and High Throughput Microplate Reader-Assisted Universal Microwell Spectrophotometric Assay for Direct Determination of Tyrosine Kinase Inhibitors in Their Pharmaceutical Formulations Irrespective the Diversity of Their Chemical Structures

**DOI:** 10.3390/molecules28104049

**Published:** 2023-05-12

**Authors:** Ibrahim A. Darwish, Nourah Z. Alzoman

**Affiliations:** Department of Pharmaceutical Chemistry, College of Pharmacy, King Saud University, P.O. Box 2457, Riyadh 11451, Saudi Arabia

**Keywords:** tyrosine kinase inhibitors, ultraviolet light absorption, microwell spectrophotometric assay, green analytical approach, high-throughput pharmaceutical analysis

## Abstract

This study discusses the development and validation of a universal microwell spectrophotometric assay for TKIs, regardless of the diversity in their chemical structures. The assay depends on directly measuring the native ultraviolet light (UV) absorption of TKIs. The assay was carried out using UV-transparent 96-microwell plates and the absorbance signals were measured by a microplate reader at 230 nm, at which all TKIs had light absorption. Beer’s law correlating the absorbances of TKIs with their corresponding concentrations was obeyed in the range of 2–160 µg mL^–1^ with excellent correlation coefficients (0.9991–0.9997). The limits of detection and limits quantitation were in the ranges of 0.56–5.21 and 1.69–15.78 µg mL^–1^, respectively. The proposed assay showed high precision as the values of the relative standard deviations for the intra- and inter-assay precisions did not exceed 2.03 and 2.14%, respectively. The accuracy of the assay was proven as the recovery values were in the range of 97.8–102.9% (±0.8–2.4%). The proposed assay was successfully applied to the quantitation of all TKIs in their pharmaceutical formulations (tablets) with reliable results in terms of high accuracy and precision. The assay greenness was evaluated, and the results proved that the assay fulfils the requirements of green analytical approach. The proposed assay is the first assay that can analyse all TKIs on a single assay system without chemical derivatization or modifications in the detection wavelength. In addition, the simple and simultaneous handling of a large number of samples as a batch using micro-volumes of samples gave the assay the advantage of high throughput analysis, which is a serious demand in the pharmaceutical industry.

## 1. Introduction

Cancer is the second leading global cause of death among men and women. In 2022, the estimated number of new cancer cases and deaths in the United States was ~1.9 million and 609,360, respectively. Cancer deaths are expected to increase significantly worldwide, with ~13.1 million deaths in 2030 [1]. Many studies documented that cancer is today’s serious problem and rapidly spreading worldwide, causing marvellous burden on communities and negatively affecting healthcare systems, particularly those in developing countries [2,3,4,5,6]. Chemotherapy is the most important option for the treatment of cancers, particularly the systemic type. Chemotherapeutic drugs usually exert their cytotoxic effects by disrupting the synthesis or the function of certain vital proteins and other cellular biomolecules involved in the pathogenesis of cancers. These drugs, despite their potent cytotoxic effects, show some major side/toxic effects because of their limited selectivity to cancer cells or tissues [7]. In recent years and because of great developments in technologies of drug discovery, new chemotherapeutic drugs with high selectivity for cellular targets onto cancer cells were discovered [8]. These new specific targeted anticancer drugs showed successful potent efficacy with no or at least minimum tolerable side effects [9]. Tyrosine kinase inhibitors (TKIs) are the most important family of these new targeted anticancer drugs [10,11,12,13].

The Food and Drug Administration (FDA) has approved more than 20 members of this family, and the approval is continuing for new members [14,15,16,17]. TKIs exert their action by attacking the tyrosine kinase enzyme which is essential for cell proliferation and molecular pathways [13]. TKIs showed high therapeutic effectiveness and improved management of different types of cancer with low side effects [10,11,12,13,14,15,16,17]. These therapeutic efficacy and safety benefits of TKIs are principally dependent on the quality of their pharmaceutical formulations in terms of their active drug contents. To ensure the accurate contents of TKIs in the formulations, a proper quantitative analytical technique is required. The analytical techniques existing in the literature for the quantitative determination of TKIs in their formulations are chromatography [18,19,20,21,22,23,24,25,26,27], voltammetry [28], spectrofluorometry [29,30,31,32], and spectrophotometry [33,34,35,36,37,38,39,40,41,42,43,44]. Among these techniques, spectrophotometry is the most convenient and widely applied technique, as evident from the number of publications [33,34,35,36,37,38,39,40,41,42,43,44]. However, all the reported spectrophotometric assays were developed individually; one assay was for one particular TKI drug. This approach was followed because of the diversity in chemical structures of TKIs. In addition, these assays suffer from major drawbacks such as laborious extraction steps [40,41,42] and consumption of large volumes of organic solvents [43]. Many studies confirmed the strong link between the practice of analysis in laboratories and the exposure of working personnel and environment to the organic solvents and the consequent occurrence of dose-dependent harmful side effects [44,45,46,47,48]. Furthermore, all these assays are conducted in the conventional manual manner, which has limited analytical throughput. Therefore, these assays neither apply the principles of the green analytical chemistry (GAC) approach [49] nor meet the needs of pharmaceutical industries to straightforward assays with high throughput for rapid analysis of large numbers of pharmaceutical samples [50]. For these reasons, the development of a universal spectrophotometric assay for quantitation of all TKIs irrespective of their different chemical structure, and overcoming these drawbacks, would be very valuable. In a previous study, Darwish et al. [51] developed a universal spectrophotometric assay for TKIs; however, the assay is still ever suffering from the use of health/environment hazardous derivatizing reagents.

**Figure 1:**
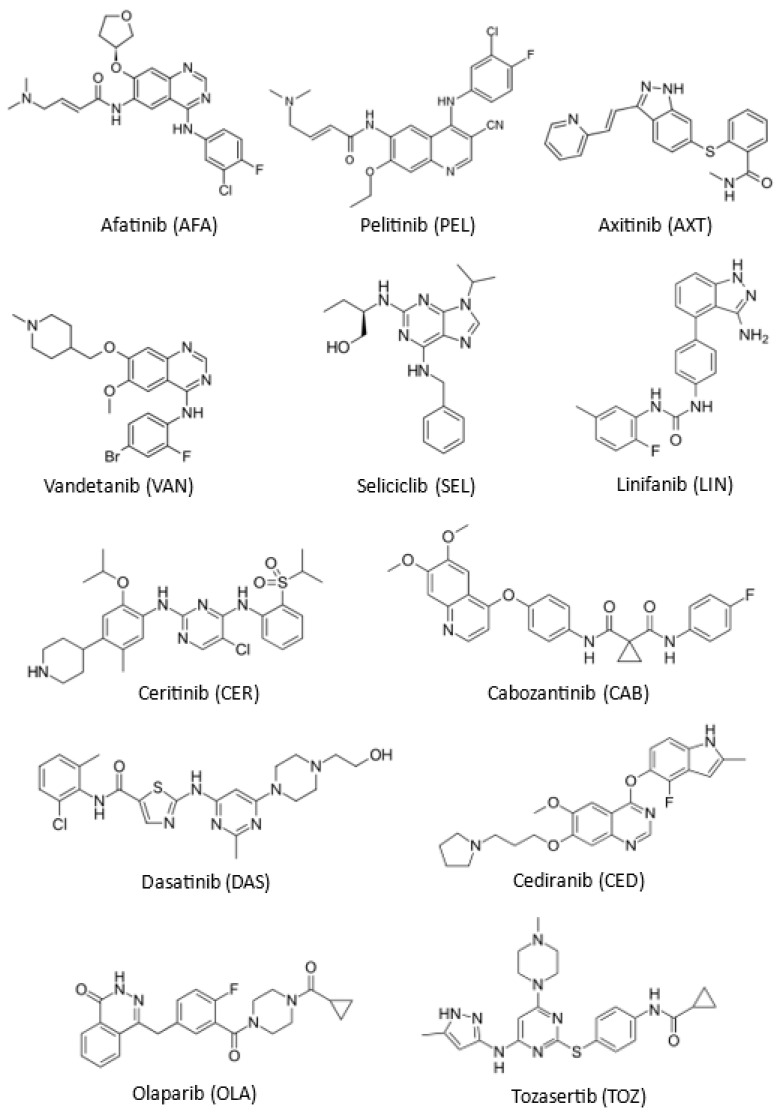
The chemical structures of the investigated tyrosine kinase inhibitors (TKIs) with their abbreviations.

The present study describes the development and validation of a universal microwell spectrophotometric assay for all TKIs, irrespective of the diversity in their chemical structure, and devoid of the previous drawbacks. The assay depends on the direct measurement of the native ultraviolet (UV) light absorption of TKIs. The assay was conducted using transparent 96-microwell plates and the UV-light absorbances were measured by an absorbance microplate reader at a single wavelength at which all TKIs had light absorption. The assay, hereafter abbreviated as MW-UV-SPA (microwell-UV-based spectrophotometric assay), was developed and validated for 12 TKIs; their chemical structures are given in Figure 1, and their chemical names, molecular formulae, and molecular weights are given in Table 1. The proposed MW-UV-SPA meets the principles of the GAC approach and fulfils the demands of high throughput analysis for the pharmaceutical industry.

## 2. Results and Discussion

### 2.1. Strategy for Assay Development

**Figure 2:**
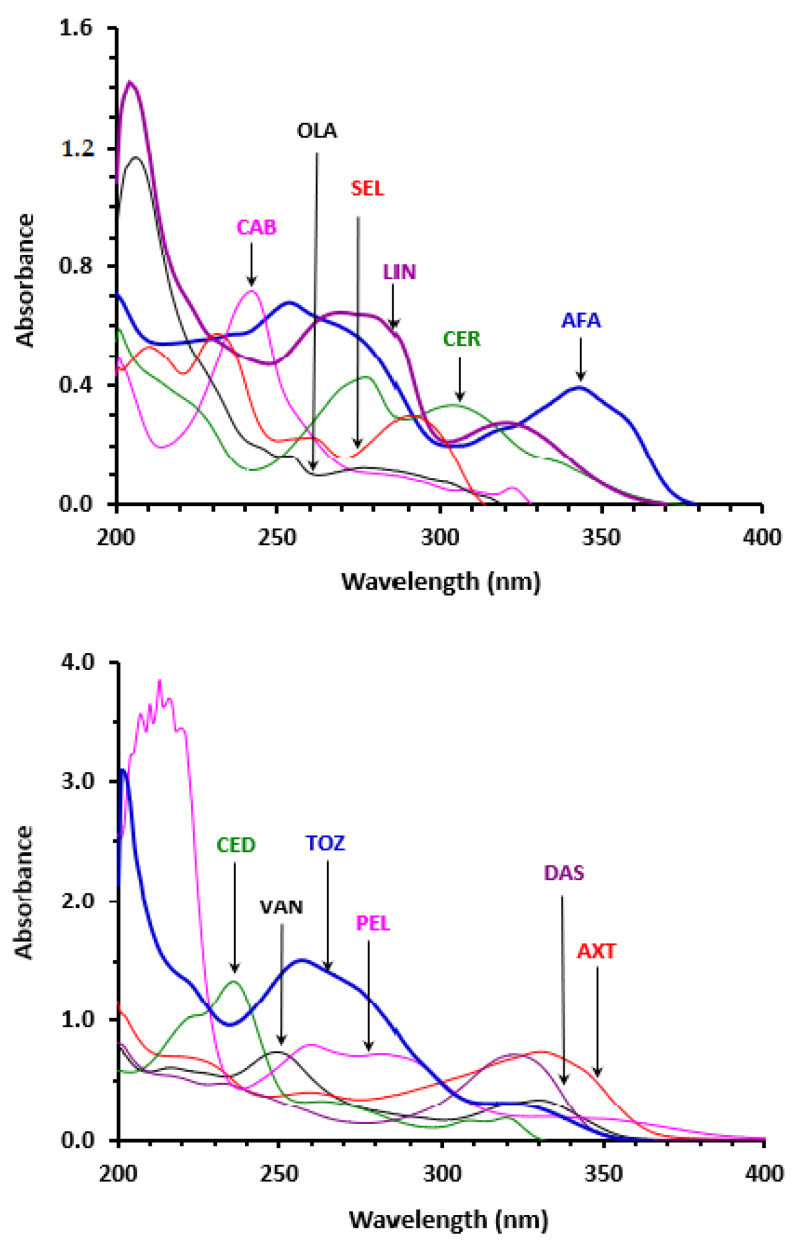
The absorption spectra of TKIs (10 µg mL^−1^, in methanol) against methanol.

Spectrophotometric assays have substantial importance and are widely used in the quality control of pharmaceuticals [52,53,54]. These assays gained their importance because they are ready for automation with spectrophotometric analyzers which enable the processing of many samples, particularly in the assessment content uniformity and studying the dissolution characteristics of solid formulations. The chemical structures of all TKIs contain aromatic conjugated chromophoric moieties which are expected to have UV-light absorption ability (Figure 1), and consequently, the development of a spectrophotometric assay for TKIs based on their UV-light absorption. This assumption was also supported by previous reports for some TKIs [33,34,35,36,37,38,39], and practically confirmed in our laboratory for all TKIs, as evident from the UV-spectra of TKIs (Figure 2). It is well established that UV-spectrophotometric assays have major advantages which include: (1) the technique is non-destructive to the sample, allowing its reused for further processing or analyses, particularly when the sample size is small or cannot be repeated, (2) measurements of absorbances can be made quickly, leading to rapid analysis, (3) instruments are usually requiring no or minimum user training because they are easy to run and analyzing data, (4) the instruments are usually inexpensive to acquire and operate, making it accessible for most pharmaceutical quality control laboratories, (5) the assays are extremely accurate and give highly precise results. For these reasons, the development of a UV-based spectrophotometric assay was considered in this study.

The reported UV-based spectrophotometric assays for some members of TKIs [33,34,35,36,37,38,39] involved the conventional manual analytical practice which employs volumetric flasks/cuvettes in the analysis. Accordingly, these assays do not fulfil the demands of pharmaceutical quality control laboratories for high throughput analysis [55,56]. Also, these assays consume large volumes of samples, mostly in costly organic solvents; therefore, these assays do not apply the principles of GAC [57,58,59]. Therefore, the present study was dedicated to the development of new alternative UV-based assays for TKIs with higher throughput and applying the principles of GAC.

Spectrophotometric assays-assisted with absorbance microplate readers are valuably used to improve the routine laboratory processes and efficiency in pharmaceutical industries (e.g., drug discovery and quality control). A microplate reader can handle up to 3456 samples in minutes or even seconds and greatly expand throughput. It also helps to save reagent costs and minimizes operational time, allowing the analyst to focus more on data analysis and the generation of valuable insights. In recent years, our laboratory developed many successful microplate readers-assisted spectrophotometric assays for different drugs [60,61,62]. These assays had high analytical throughput and apply the principles of GAC. Therefore, the present study was dedicated to employing an absorbance microplate reader in the development of microwell-UV-based spectrophotometric assay (MW-UV-SPA) for TKIs.

### 2.2. Development of MW-UV-SPA

#### 2.2.1. UV-Absorption Spectra and Selection of Proper Wavelength for Measurement

The UV-absorption spectra of the 12 cited TKIs were recorded in the range of 200–400 nm (Figure 2). The spectra, as expected from their different chemical structures, varied in their shapes, maximum absorption peaks (λ_max_) and molar absorptivities (ε). The λ_max_ and ε values of all TKIs are summarized in Table 2. At λ_max_, PEL had the highest ε value, followed by TOZ and CED. Also, the λ_max_ of LIN and OLA were highly blue-shift (at 204 and 201 nm, respectively) and appeared before the absorption cut-off of the solvent (205 nm); therefore, these λ_max_ were not practical in analytical terms, and thus they were ruled out form further considerations.

The main goal of the present study was the development of a universal assay for all TKIs, irrespective of their different chemical structures. To achieve this goal, it was necessary to carefully select a wavelength at which all TKIs have UV-light absorption with ε values adequate for the development of a sensitive assay of all TKIs. Upon inspection of the spectra overall the full spectrum range, it was found that all TKIs have UV-light absorption at 230 nm (Figure 2). At 230 nm, the ε values were recalculated, and the obtained values are summarized in Table 2. Obviously, all the ε values are adequately high for enabling a sensitive universal assay for all TKIs in their pharmaceutical formulations. According to these findings, further investigations were conducted at 230 nm.

#### 2.2.2. Optimization of Assay Conditions

The conditions of the proposed MW-UV-SPA were optimized for selecting the most appropriate solvent, pH of the solution, and volume of standard or sample solution. Different solvents (water, methanol, ethanol, and acetonitrile) were used for preparing the working TKIs solutions. The results showed slight effects for the solvents on ε values of most TKIs. In the case of PEL, DAS, and TOZ, aqueous solutions turned slightly opalescent because of their low solubility in water. Since the study was directed to develop a universal assay for all TKIs, methanol was used as a solvent for the subsequent experiments. The ε values were also determined for the TKIs working solutions in buffered systems of varying pH values (pH 3–10). It was observed that the acidic pH values (pH 3–5) had variable negative effects on the ε values of TKI solutions. These effects were probably due to the quaternization of amino groups in the chemical structures of all TKIs [52]. The ε values achieved in buffered solutions of pH values in the range of 6–8 were the same as those obtained for non-buffered aqueous solutions. Solutions of pH values of 9–10 showed lower ε values with most TKIs, which may probably be due to the instability of the TKIs in these alkaline solutions. According to these findings, non-buffered aqueous solutions were used for subsequent experiments.

**Figure 3:**
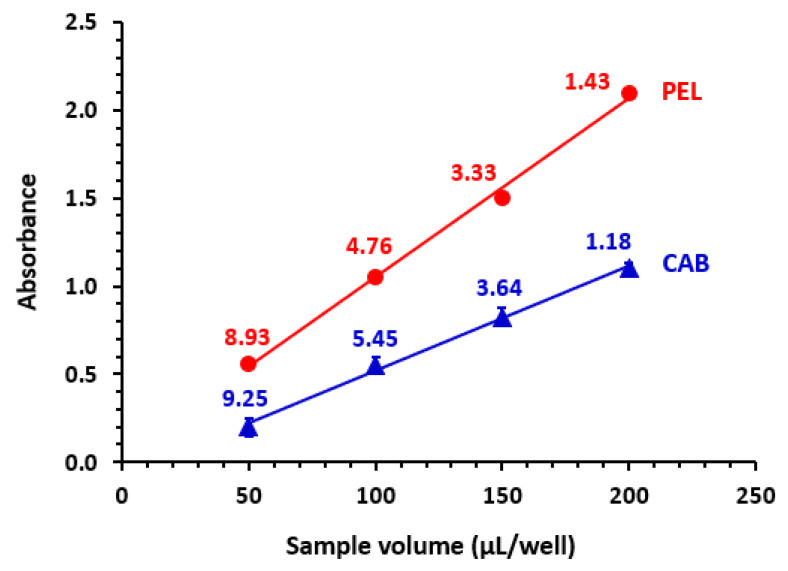
Effect of volume of TKI solution (50 µg mL^−1^) on the absorbance measured by the absorbance microwell reader. The values are mean of 3 determinations ± SD and the figure given on each point is the relative standard deviation of the readings.

Since the microplate reader measures the absorbance of the test solution in the assay plate wells by passing light vertically through each well, the measured absorbance depends on the volume of the sample in the well, which is the actual light pathlength. Therefore, it was necessary to study the most appropriate volume of TKI solution which could be dispensed into each well of the assay plate. Different volumes (50, 100, 150, and 200 µL) of each TKI solution were dispensed in each well. The absorbances were measured, and the RSD values of the readings were calculated for each volume to assess the readings precision. It was found that the absorbance increases linearly with the volume of test TKI solution. Also, it was found that the RSD values decrease as the volumes of solution increase. The lowest RSD values (highest precision) were achieved when 200 µL/well was used, and the RSD values were <2% for all TKIs. The results are given for CAB and PEL, as representative examples (Figure 3), and similar results were obtained for all TKIs. According to these findings, all the subsequent experiments were conducted using 200 µL of TKI solution per well of the assay plate.

A summary of the optimization of assay conditions and the optimum value/condition are summarized in Table 3. Under these conditions, the assay was validated [63].

### 2.3. Validation of MW-UV-SPA

#### 2.3.1. Linear Range and Sensitivity

**Figure 4:**
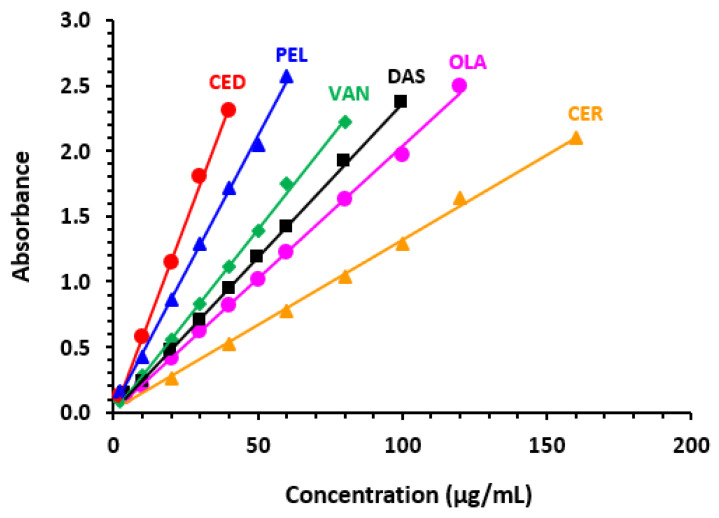

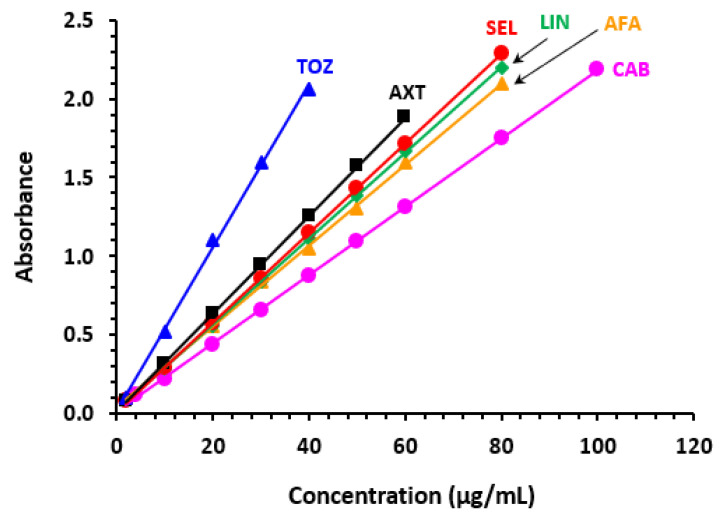
Calibration curves for quantitation of TKIs by the proposed microwell UV-SFA. Values of absorbance are the mean of 5 determinations.

Under the established optimum conditions of the MW-UV-SPA (Table 3), the calibration curves were constructed for all TKIs (Figure 4), and the results of the linear regression analysis of the data are summarized in Table 4. The curves were linear with small intercepts and excellent correlation coefficients in a general range of 2–160 μg mL^−1^. It is worth mentioning that we did not use TKI concentrations giving absorbances more than 2.5 to avoid any potential negative effect of stray light on the linearity of the assay, even the absorbance plate readers typically incorporate features for minimizing stray light effects. The values of LOD and LOQ were in the range of 0.56–5.21 and 1.69–15.78 μg mL^−1^, respectively. A summary of the calibration and validation parameters of the proposed MW-UV-SPA is given in Table 4.

#### 2.3.2. Precision and Accuracy

The results of precision assessment revealed the high intra- and inter-assay precisions of the proposed MW-UV-SPA for TKIs as evidenced from the low values of RSD which did not exceed 2.03 and 2.14%, respectively (Table 5). This high level of precision was attributed to two main reasons. The first one is the uniformity in the sample volumes from well to well in the assay plate. This uniformity arises from the precise simultaneous dispensing of solutions by the multi-channel pipette. The second reason is the direct measurement of the absorbances without any extra experimental manipulations (e.g., the addition of reagent, derivatization reaction, etc.) which could negatively affect the assay precision.

The results of recovery studies confirmed the accuracy of the proposed MW-UV-SPA for TKIs as the recovery values were ≥97.8 (Table 5).

#### 2.3.3. Selectivity

The selectivity of the proposed MW-UV-SPA for the accurate quantitation of TKIs was assessed by recovery studies for known quantities of TKIs mixed with pharmaceutical excipients. The obtained recovery values for all TKIs with the tested excipients were in the range of 98.4–103.5% ± 0.8–2.2% (Table 6). These high recovery values proved the selectivity of the proposed assay for the quantitation of TKIs in their pharmaceutical formulations without any interferences from the formulation excipients. The absence of interference from excipients was due to the extraction of TKI from the samples by methanol in which the excipients did not dissolve.

#### 2.3.4. Robustness and Ruggedness

The proposed MW-UV-SPA involves the direct measurement of the native UV-light absorption of TKIs without any experimental manipulations; therefore, is inherently robust. The ruggedness of the assay was assessed by applying the assay to the analysis of the investigated TKIs by two different analysts at two different laboratories at different elapsed times. The results confirmed the ruggedness of the assay as the RSD values were ≥97.8%.

### 2.4. Application of MW-UV-SPA to the Analysis of Pharmaceutical Formulations

The satisfactory results of the assay validation on the bulk form of TKIs indicated the suitability of the assay for routine analysis of pharmaceutical formulations (tablets) of TKIs. Upon application of the assay to the analysis of different pharmaceutical formulations, the assay gave very good results. The obtained mean values of the labelled amounts ranged from 98.9 ± 2.1% to 102.1 ± 2.4% (Table 7).

### 2.5. Greenness of MW-UV-SPA

Basically, the microwell-based assays using microplate readers for measuring the signals mostly fulfill the requirements of GAC practice. This fulfillment was achieved because these assays use small volumes of samples, reagents, and yield minimal waste. For a precise assessment of the greenness of the proposed MW-UV-SPA for TKIs, four different metric tools were used. These tools were the NEMI [64], ESA [65], GAPI [66], and AGREE [67]. The procedure of greenness assessment by these tools was given in the experimental section.

**Figure 5:**
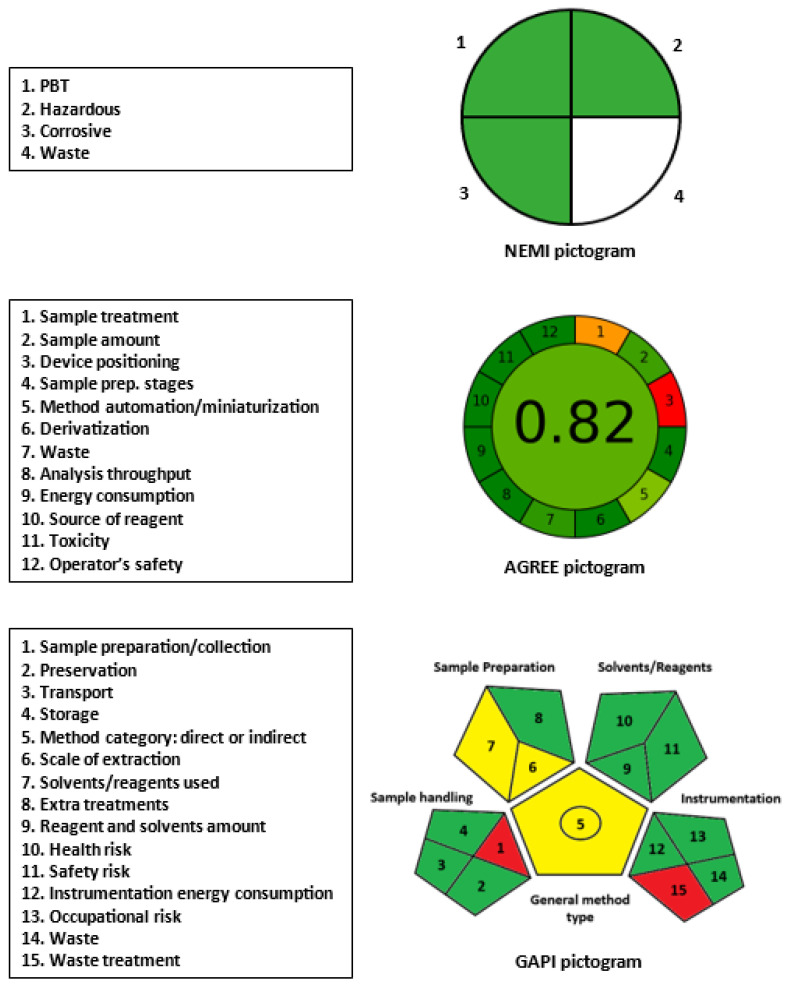
Results of NEMI, AGREE, and GAPI analysis for evaluation of the greenness of the proposed microwell UV-SFA for TKIs.

In NEMI, the four categories of greenness assessment (PBT, hazardous, waste and corrosive) are green (Figure 5). The quadrant corresponding to the waste category did not take a green color because of wasting methanol, even using a small volume (200 µL) per sample. In ESA, the total PPs of the assay were 9, and accordingly, the total eco-score of the assay was 92 out of 100 (Table 8). Upon applying AGREE tool, parameter number 1 (sample treatment) took orange color because the sample treatment was not carried out online; however, it was carried out offline with few steps. Also, parameter number 3 (device positioning) took red color because the analysis on the microplate reader was not carried out in an automated manner. The generated number appeared in the centre of the AGREE’s pictogram, which indicates an overall acceptance of 0.82 out of 1 (Figure 5). The results of GAPI tools (Figure 5) indicated that two parameters took red color; these parameters were the sample preparation/collection (1) and waste treatment (15). This result was due to the offline sample preparation/collection and wasting methanol, respectively. The other parameters were mostly green.

In conclusion, the overall evaluation of the eco-friendly and greenness of the proposed MW-UV-SPA fulfills the requirements for GAC for routine use in pharmaceutical quality control laboratories for analysis of TKIs.

### 2.6. Advantages of the Proposed Assay over the Previous Assays

The proposed MW-UV-SPA has advantages over the previously published assays for TKIs. These advantages are summarized in the following points: (1) The proposed assay is the first assay that can analyze all TKIs on a single assay system without chemical derivatization or modifications in the detection wavelength. (2) The proposed assay employed the one-step direct measurements of native UV absorption of TKIs, rather than the previous assays those involved chemical derivatization steps. (3) The proposed assay involved small sample volumes for measurements which saves costs and made the assay procedures green. (4) The proposed assay has higher throughput than the previously reported assay.

## 3. Experimental

### 3.1. Instruments

Microplate reader (SpectraMax^®^ M5: Molecular Devices, LLC., San Jose, CA, USA) with multi-mode detections (UV-visible absorbance, chemiluminescence, fluorescence, fluorescence polarization, and time-resolved fluorescence). The reader features include a triple-mode cuvette port, spectral scanning in 1 nm increments, and up to six wavelengths per read. It is equipped with a dual scanning monochromator optics that allows the reader to read in a wavelength range of 200 to 1000 nm, all without the need to change filters. The reader uses standard 6-well to 384-well microplates to read endpoint, kinetic, spectrum, and multi-point well-scanning in absorbance and fluorescence using cuvettes. The reader is also equipped with a 4-Zone temperature control system, allowing for temperature control up to 50 °C that provides excellent stability for temperature-sensitive assays. An internal shaker with variable speeds (low, medium, and high) is also featured on the reader. SpectraMax M5 reader utilizes SoftMax^®^ Pro Enterprise software, version 7.2 GxP (Molecular Devices, LLC., San Jose, CA, USA), the industry-leading data acquisition and analysis software with FDA 21 CFR Part 11 compliance tools. Microprocessor laboratory pH meter (BT-500: Boeco, Hamburg, Germany), digital balance (JB1603-C/FACT: Mettler-Toledo International Inc., Zürich, Switzerland).

### 3.2. Materials, Tools and Pharmaceutical Formulations

Standard materials of TKIs were purchased from LC Laboratories (Woburn, MA, USA); their purities were >99%, and used as received. UV-transparent 96-microwell plates (Cat No. CLS 3635) were purchased from Corning/Costar Inc. (Cambridge, MA, USA). Variable volumes and adjustable multi-channel-pipettes were products of Sigma-Aldrich Chemicals Co. (St. Louis, MO, USA). Spectroscopic grade methanol and other solvents were obtained from Fisher Scientific (California, CA, USA). All other reagents were of analytical grade from different suppliers. The pharmaceutical formulations of TKIs used in this study with their manufacturers and strength are given in Table 9.

### 3.3. Preparation of Standard TKI Solution

Stock standard solutions (2 mg mL^−1^) of TKIs (except PEL, DAS and TOZ) were prepared by dissolving 40 mg of the standard material of TKI in 20 mL with methanol. Standard solutions of PEL, DAS, and TOZ, because of their low solubility, were prepared at a concentration of 0.5 mg mL^−1^ by dissolving 10 mg of each of these TKIs in 20 mL methanol. Stock solutions were stable for 14 days on storage in a refrigerator (8 °C). Working solutions of all TKIs were prepared at a concentration of 200 μg mL^−1^ by diluting proper volumes of the stock solutions with methanol.

### 3.4. Preparation of Pharmaceutical Formulation Solution

Ten tablets of each TKI tablet were finely pulverized and amounts equivalent to 40 mg of each of TKIs (except PEL, DAS and TOZ) were accurately weighed and transferred into a 20 mL volumetric flask. In the case of PEL, DAS and TOZ, 20 mg was transferred. The contents of the flasks were dissolved in ~15 mL of methanol by sonication for 30 min in an ultrasonic bath. The volumes were completed up with the same solvent (methanol), mixed well, and then settled for approximately 15 min before filtering off the solutions. The first portion of each solution was discarded, and the supernatants were diluted with methanol to give final concentrations of 200 μg mL^−1^, for each formulation of the cited TKIs. These solutions were subjected to analysis for their contents of TKIs by the proposed MW-UV-SPA.

### 3.5. Procedure of MW-UV-SPA and Construction of Calibration Curves

Aliquots (200 µL) of the working TKI solutions containing varying concentrations of the cited TKIs were transferred into each well of the assay plate. Blank wells received 200 µL of methanol instead of TKI samples. The absorbance of each well was measured by the microplate reader at 230 nm. The measured absorbances, after subtracting those of blank wells, were plotted against the corresponding concentrations of TKIs to construct the calibration curves of TKIs. Regression analysis for the calibration graphs data was conducted and linear fitting equations were derived along with their parameters (intercepts, standard deviations of intercepts, slopes, standard deviations of slopes, and correlation coefficients). The linear fitting equations were used for the determination of TKIs contents in their sample formulations.

### 3.6. Validation of MW-UV-SPA Procedure

The analytical performance of the proposed MW-UV-SPA was validated according to the guidelines of the International Council of Harmonization (ICH) for validation of the analytical procedure [63]. The validation of the assay was conducted in terms of its linearity range, sensitivity, precision, accuracy, selectivity, robustness, and ruggedness.

#### 3.6.1. Assessment of Linearity Range and Sensitivity

Linearity was assessed by generating calibration curves using 5 replicates of at least 5 concentration levels of each TKI. The absorbance-concentration data was subjected to regression analysis and the linear fitting equations were derived along with the parameters of the equations. These parameters were intercept (a), standard deviation of the intercept (SDa), slope (b), standard deviation of the slope (SDb) and correlation coefficient (r). The value of the correlation coefficient of each calibration line was used as a measure for the assay linearity. The sensitivity of the assay for each TKI was expressed as the limit of detection (LOD) and limit of quantitation (LOQ). LOD and LOQ were calculated by using the formula: LOD or LOQ = (f × SDa)/b, where f was a factor of 3.3 and 10 for LOD and LOQ, respectively.

#### 3.6.2. Estimation of Precision and Accuracy

The intra-assay precision of the proposed MW-UV-SPA was estimated by analysis of 5 replicate samples (*n* = 5) of each TKI solution as a batch in a single assay run. The inter-assay precision was estimated by analysis of 3 replicate samples (*n* = 3) of each TKI solution on 2 consecutive days. The relative standard deviation (RSD, expressed as %) was used as a measure for the estimation of precision level.

The accuracy of the proposed MW-UV-SPA for each TKI was estimated by conducting recovery studies. The known nominated concentration of each TKI solution was subjected to the analysis by the proposed assay, and the concentration was determined using the linear calibration equation (measured concentration). Recovery, expressed as %, was calculated by the formula: Recovery (%) = (measured concentration/nominated concentration) × 100.

#### 3.6.3. Evaluation of Selectivity

The selectivity of the proposed MW-UV-SPA for accurate quantitation of the TKIs without any potential interferences from the inactive ingredients (excipients) that are used for the pharmaceutical formulations of TKIs was evaluated. Test samples were prepared by mixing a known amount (50 mg) of each TKI with different amounts of excipients. These excipients were microcrystalline cellulose (MCC), starch, lactose monohydrate (LMH) and magnesium stearate (MS). The amounts used for these excipients were 50, 10, 5, and 5 mg for MCC, starch, LMH, and MS, respectively. The prepared samples were treated as described under the section “preparation of pharmaceutical formulation solution”. The treated solutions were analyzed by the proposed assay and their contents of TKIs. Recovery values were determined and used as measures for selectivity.

#### 3.6.4. Assessment of Robustness and Ruggedness

The assay robustness is defined as the effect of minor changes in the experimental assay’s procedure on its reliability (accuracy and precision). Since the proposed MW-UV-SPA is based on a direct measurement of the native UV-light absorption of TKIs without any experimental variables, the assay was considered robust.

The assay ruggedness is a measure of reproducibility of assay results obtained under different operating assay conditions normally from laboratory to laboratory and analyst to analyst. The ruggedness of the proposed MW-UV-SPA was assessed by applying the assay to the analysis of the investigated TKIs by two different analysts at two different laboratories at different elapsed times. The obtained results were expressed as RSD (%).

### 3.7. Greenness Assessment Procedures

Four tools were used for the assessment of the MW-UV-SPA greenness; these tools and their procedure are summarized in the following sections.

#### 3.7.1. National Environmental Method Index

The national environmental method index (NEMI) is the first tool used to assess the impact of an analytical procedures on the environment [64]. The NEMI tool categorizes the greenness parameters into four categories, presented as four quadrants in a circular pictogram. The parameters of the four categories (chemicals of certain characteristics, pH values, and waste) were simulated in the circular pictogram of four quadrants. These four categories are PBT, hazardous, corrosive, and waste. PBT is a persistent, bio-accumulative, and toxic substances involved in the assay procedure. Each category appears in its particular quadrant as either blank or green, depending on matching the requirements or not. The overall assessment of the assay greenness can be done easily by the analyst, through visual inspection of the pictogram.

#### 3.7.2. Eco-Scale Assessment Tool

The eco-scale assessment (ESA) tool was proposed by Gałuszka et al. [65]. ESA tool classifies the greenness of an assay procedure into four categories. These categories are the reagents used in the assay, energy consumption by the analytical instruments, occupational hazards and waste. Each category is given penalty points (PPs) according to their harmful impact on the environment. The total PPs of the assay categories are subtracted from a total score of 100 points (the total score of an ideal green assay procedure with no PPs). ESA tool classifies the analytical procedures into three greenness levels: green procedure (which has a final total score of >75 points), reasonably green procedure (which has a final total score of 50–75 points), and inadequate green procedure (which has a final score of <50 points).

#### 3.7.3. Green Analytical Procedure Index (GAPI)

The green analytical procedure index (GAPI) was invented by Płotka-Wasylka in 2018 [66]. GAPI tool is reliable and can provide a comprehensive ecological assessment of the entire analytical procedure, starting from the sample collection/preparation to the end of the analysis. The entire assessment includes 15 parameters (1–15) under five different categories (A, B, C, D and E). The details of parameters and categories are as follows:

Category A (sample handling) includes 4 parameters (1–4). 1: type of sample preparation/collection, 2: preservation, 3: transport, and, 4: storage.

Category B (method category and quantification mark) describes the method type either direct or indirect, and a circle in the middle refers to a quantitative nature of the analytical technique.

Category C (sample preparation) includes 3 parameters (6–8). 6: extraction scale, 7: solvents/reagents used, and 8: extra sample treatments.

Category D (solvents/reagents used in the analytical procedure) includes 3 parameters (9–11). 9: amounts of reagents/solvents, 10: health hazardous, and 11: safety risk.

Category D (instrumentation) includes 4 parameters (12–15). 12: energy consumption by the instruments, 13: occupational risk, 14: waste, and 15: waste treatment.

Each parameter of these 15 parameters is presented as a section of a pictogram (of 15 sections). Each section is given a color (green, yellow, or red), where the green color indicates a safe procedure while the red color refers to a non-eco-friendly procedure.

#### 3.7.4. Analytical Greenness Metric Tool (AGREE)

The analytical Greenness (AGREE) metric tool [67] is the most recent greenness assessment tool. AGREE is a comprehensive, flexible, and straightforward software (version 0.5 beta, developed by Grańsk University of Technology, Gdańsk, Poland) that provides an easily interpretable and informative results. The assessment criteria in the AGREE software are taken from the 12 principles of GAC presented in the word “SIGNIFICANCE” and are transformed into a unified 0–1 scale. The automatically generated circular pictogram is divided into 12 sections; each section has a specific color range from deep green (=1) to deep red (=0). The overall score (a fraction of unity) is automatically calculated and appears in the middle of the pictogram.

## 4. Conclusions

The present study described, for the first time, the development and validation of a universal MW-UV-SPA for 12 TKIs at a single detection wavelength, regardless of the differences in their chemical structures. The proposed assay combined many additional advantages over all the reported spectrophotometric assays for TKIs. These advantages include the simplicity of the assay procedure (convenience), consumption of low volumes of samples/solvents (economic and green approach), and high throughputs (applicable in the pharmaceutical industry). Furthermore, the assay has a high sensitivity for quantitation of low concentrations of TKIs with high accuracy and precision. In overall conclusion, the results of the present study widen the perception of the efficient and valuable employment of microwell assays assisted with microplate readers for the quantitation of TKIs and drugs in pharmaceutical industries.

## Figures and Tables

**Table 1:** The investigated TKIs with their abbreviations, IUPAC names, molecular formulae, and molecular weights.

TKI Name	Abbreviation	IUPAC Name	Molecular Formula	Molecular Weight
Afatinib	AFA	(*Z*)-but-2-enedioic acid;(*E*)-*N*-[4-(3-chloro-4-fluoroanilino)-7-[(3*S*)-oxolan-3-yl]oxyquinazolin-6-yl]-4-(dimethylamino)but-2-enamide	C_24_H_25_ClFN_5_O_3_	485.94
Pelitinib	PEL	(2*E*)-*N*-{4-[(3-chloro-4-fluorophenyl)amino]-3-cyano-7-ethoxyquinolin-6-yl}-4-(dimethylamino)but-2-enamide	C_24_H_23_ClFN_5_O_2_	467.90
Cediranib	CED	4-[(4-fluoro-2-methyl-1*H*-indol-5-yl)oxy]-6-methoxy-7-(3-pyrrolidin-1-ylpropoxy)quinazoline	C_25_H_27_FN_4_O_3_	450.51
Axitinib	AXT	*N*-methyl-2-[[3-[(*E*)-2-pyridin-2-ylethenyl]-1*H*-indazol-6-yl]sulfanyl] benzamide	C_22_H_18_N_4_OS	386.47
Ceritinib	CER	5-chloro-2-*N*-(5-methyl-4-piperidin-4-yl-2-propan-2-yloxyphenyl)-4-*N*-(2-propan-2-ylsulfonylphenyl)pyrimidine-2,4-diamine	C_28_H_36_ClN_5_O_3_S	558.14
Cabozantinib	CAB	*N*’1-{4-[(6,7-dimethoxyquinolin-4-yl)oxy]phenyl}-*N*1-(4-fluorophenyl)cyclopropane-1,1-dicarboxamide	C_28_H_24_FN_3_O_5_	501.50
Linifanib	LIN	1-[4-(3-amino-1*H*-indazol-4-yl)phenyl]-3-(2-fluoro-5-methylphenyl)urea	C_17_H_15_FN_5_O	324.34
Olaparib	OLA	4-[[3-[4-(cyclopropanecarbonyl)piperazine-1-carbonyl]-4-fluorophenyl]methyl]-2H-phthalazin-1-one	C_24_H_23_FN_4_O_3_	434.47
Seliciclib	SEL	(2*R*)-2-{[6-(benzylamino)-9-(propan-2-yl)-9*H*-purin-2-yl]amino}butan-1-ol	C_19_H_26_N_6_O	354.46
Vandetanib	VAN	*N*-(4-bromo-2-fluorophenyl)-6-methoxy-7-[(1-methylpiperidin-4-yl)methoxy]quinazolin-4-amine	C_22_H_24_BrFN_4_O_2_	475.40
Dasatinib	DAS	*N*-(2-chloro-6-methylphenyl)-2-[[6-[4-(2-hydroxyethyl)piperazin-1-yl]-2-methylpyrimidin-4-yl]amino]-1,3-thiazole-5-carboxamide	C_22_H_26_ClN_7_O_2_S	488.01
Tozasertib	TOZ	*N*-[4-[4-(4-methylpiperazin-1-yl)-6-[(5-methyl-1*H*-pyrazol-3-yl)amino] pyrimidin-2-yl]sulfanylphenyl]cyclopropanecarboxamide	C_23_H_28_N_8_OS	464.59

**Table 2:** UV-spectral data of TKIs.

TKIs	λ_max_ (nm)	Absorbance		ε × 10^4^ (L mol^−1^ cm^−1^)	
		At λ_max_	At 230 nm	At λ_max_	At 230 nm
AFA	254	0.678	0.559	3.29	2.72
PEL	213	3.854	0.437	18.00	2.19
CED	236	1.338	0.259	6.03	1.45
AXT	330	0.741	0.556	2.86	1.80
CER	277	0.426	0.408	2.38	1.77
CAB	242	0.716	0.573	3.59	2.03
LIN	204	1.414	0.555	4.59	2.64
OLA	277	0.124	0.628	0.54	2.43
SEL	232	0.577	1.153	2.05	5.19
VAN	250	0.745	0.473	3.54	2.31
DAS	322	0.716	0.859	3.49	4.02
TOZ	201	3.089	1.033	14.40	4.80

**Table 3:** Optimization of experimental conditions for the MW-UV-SPA for TKIs.

Condition	Studied Range	Optimum Value ^a^
Solvent	Different ^b^	Methanol
pH of buffer solution	3–10	Without buffer
Sample volume (µL/well)	50–200	200
Measuring wavelength (nm)	200–400	230

^a^ Optimum values were used for all TKIs. ^b^ Solvents tested were water, methanol, ethanol, and acetonitrile.

**Table 4:** Calibration parameters for the quantitation of TKIs by MW-UV-SPA.

TKIs	Linear Range ^a^	Intercept	SDa ^b^	Slope	SDb ^b^	r ^b^	LOD ^a^	LOQ ^a^
AFA	2–80	0.0123	0.0099	0.0278	0.0044	0.9994	1.18	3.56
PEL	4–100	0.0341	0.0098	0.0217	0.0067	0.9992	1.49	4.52
CED	4–160	0.0061	0.0202	0.0128	0.0092	0.9996	5.21	15.78
AXT	2–90	0.0038	0.0100	0.0274	0.0050	0.9991	1.20	3.65
CER	2–120	0.0213	0.0099	0.0200	0.0021	0.9994	1.63	4.95
CAB	2–80	0.0130	0.0099	0.0279	0.0035	0.9995	1.17	3.55
LIN	2–80	0.0196	0.0099	0.0275	0.0044	0.9995	1.19	3.60
OLA	2–80	0.0281	0.0098	0.0313	0.0032	0.9991	1.03	3.13
SEL	2–40	0.0423	0.0097	0.0575	0.0045	0.9992	0.56	1.69
VAN	4–100	0.0115	0.0099	0.0233	0.0044	0.9994	1.40	4.25
DAS	2–60	0.0256	0.0098	0.0422	0.0037	0.9997	0.77	2.32
TOZ	2–50	0.0629	0.0097	0.0500	0.0080	0.9992	0.64	1.94

^a^ Values are in µg mL^−1^. ^b^ SDa = standard deviation of the intercept, SDb = standard deviation of the slope, r = correlation coefficient.

**Table 5:** Precision and accuracy of the proposed MW-UV-SPA for quantitation of TKIs.

TKIs	Relative Standard Deviation (%)		Recovery (% ± SD) ^c^
	Intra−Assay (*n* = 5) ^a^	Inter−Assay (*n* = 6) ^b^	
AFA	0.64	1.54	100.4 ± 1.4
PEL	1.24	2.13	101.2 ± 0.8
CED	1.62	1.85	99.4 ± 1.2
AXT	1.05	1.62	101.8 ± 1.5
CER	1.12	1.82	102.9 ± 1.1
CAB	2.03	2.14	97.8 ± 2.4
LIN	1.42	1.42	99.4 ± 2.2
OLA	1.32	1.24	101.5 ± 2.1
SEL	1.51	0.89	99.6 ± 1.2
VAN	1.42	1.52	98.5 ± 2.1
DAS	1.15	2.13	102.8 ± 1.6
TOZ	1.82	1.72	101.9 ± 1.4

^a^ The 5 replicates were analyzed as a batch in a single run. ^b^ Three replicates were analyzed on two consecutive days. ^c^ Values are mean of three determinations.

**Table 6:** Analysis of TKIs in the presence of the excipients that are present in their pharmaceutical tablets by the proposed microwell UV-SPA.

TKI	Recovery (% ± SD) ^a^			
	MCC (50) ^b^	Starch (10) ^b^	LMH (5) ^b^	MS (5) ^b^
AFA	100.2 ± 1.4	99.2 ± 1.2	99.4 ± 0.8	102.6 ± 1.3
PEL	101.4 ± 1.2	101.4 ± 1.9	100.2 ± 1.2	99.4 ± 1.4
CED	100.5 ± 0.8	99.5 ± 1.2	99.6 ± 0.9	100.1 ± 1.5
AXT	102.2 ± 1.6	101.4 ± 1.6	98.5 ± 2.1	103.2 ± 0.9
CER	100.4 ± 1.4	103.5 ± 2.2	103.1 ± 1.2	99.3 ± 1.6
CAB	99.3 ± 1.7	99.2 ± 1.4	99.4 ± 1.4	101.2 ± 1.2
LIN	101.8 ± 1.3	102.4 ± 1.5	102.1 ± 1.5	98.8 ± 1.8
OLA	100.5 ± 0.9	100.6 ± 1.2	101.2 ± 1.2	102.5 ± 1.6
SEL	98.6 ± 1.2	98.5 ± 0.8	100.3 ± 0.8	100.8 ± 1.2
VAN	99.4 ± 1.8	101.8 ± 1.4	98.8 ± 1.4	99.6 ± 2.1
DAS	103.4 ± 1.1	98.9 ± 1.2	102.5 ± 1.5	102.6 ± 1.2
TOZ	98.4 ± 1.4	97.5 ± 2.1	100.8 ± 1.6	99.4 ± 1.5

^a^ Values are mean of three determinations. ^b^ Abbreviations are: MCC = microcrystalline cellulose, LMH = lactose monohydrate, MS = Magnesium stearate. Figures in parenthesis are the amounts in mg added per 50 mg of TKI.

**Table 7:** Results of analysis of pharmaceutical tablets containing TKIs by the proposed microwell UV-SPA.

Tablets (TKI, mg/Tablet)	Label Claim (% ± SD)
Inlyta (AXT, 50)	102.1 ± 2.4
Recentin (CED, 30)	99.8 ± 1.2
Gilotrif (AFA, 40)	100.8 ± 2.4
Cabometyx (CAB, 40)	101.2 ± 1.6
Caprelsa (VAN, 300)	100.4 ± 1.8
Sprycel (DAS, 50)	98.9 ± 2.1
Lynparza (OLA, 150)	100.5 ± 1.5
LM-Ceritinib (CER, 50)	101.2 ± 2.1
LM-Linifanib (LIN, 25)	99.6 ± 1.8
LM-Tozasertib (TOZ, 100)	101.8 ± 1.6

**Table 8:** Results of Eco-Scale assessing the greenness of the proposed MW-UV-SPA for the quantitation of TKIs.

Eco-Scale Score Parameters	Penalty Points (PPs)
Reagents/solvent	
Methanol	6
	∑ = 6
Instrument: Energy used (kWh per sample)	
Microplate reader	0
pH meter	0
Vortex mixer	0
Sonicator	0
Centrifuge	0
	∑ = 0
Occupational hazardous	
Analytical process hermetic	0
Emission of vapors and gases to the air	0
	∑ = 0
Waste	
Production (<1 mL (g) per sample)	0
Treatment (No treatment involved)	3
	∑ = 3
Total PPs	9
Total Eco-Scale score	92

**Table 9:** The pharmaceutical formulations (tablets) used in the study.

Trade Name of Tablets	TKI	Strength (mg/Tablet)	Manufacturer (Address)
Inlyta	AXT	50	Pfizer (New York, NY USA)
Recentin	CED	30	AstraZeneca (Cambridge, UK)
Gilotrif	AFA	40	Boehringer Ingelheim (Ingelheim am Rhein, Germany)
Cabometyx	CAB	40	Exelixis, Inc. (Alameda, CA, USA)
Caprelsa	VAN	300	(AstraZeneca, Cambridge, UK)
Sprycel	DAS	50	(Bristol Myers Squibb, New York, NY, USA)
Lynparza	OLA	150	(AstraZeneca, Cambridge, UK))
Ceritinib	CED	50	Lab-made: Prepared in the laboratory ^a^
Linifanib	LIN	25	Lab-made: Prepared in the laboratory ^a^
Tozasertib	TOZ	100	Lab-made: Prepared in the laboratory ^a^

^a^ Tablets were prepared by mixing accurate dose with 25 mg of starch, hydroxypropyl cellulose, microcrystalline cellulose, and lactose monohydrate.

## Data Availability

All data are available in the article.

## References

[1] World Health Organization, Geneva, Report 12 September 2018. https://www.who.int/news-room/fact-sheets/detail/cancer.

[2] Milaat W.A. (2000). Knowledge of secondary-school female students on breast cancer and breast self-examination in Jeddah, Saudi Arabia. East. Mediterr. Health J..

[3] Ezzat A.A., Ibrahim E.M., Raja M.A., Al-Sobhi S., Rostom A., Stuart R.K. (1999). Locally advanced breast cancer in Saudi Arabia: High frequency of stage III in a young population. Med. Oncol..

[4] Hashim T.J. (2000). Adolescents and cancer: A survey of knowledge and attitudes about cancer in eastern province of Saudi Arabia. J. Fam. Community Med..

[5] Prager G.W., Braga S., Bystricky B., Qvortrup C., Criscitiello C., Esin E., Sonke G.S., Martínez G., Frenel J.S., Karamouzis M. (2018). Global cancer control: Responding to the growing burden, rising costs and inequalities in access. ESMO Open.

[6] Nwagbara U.I., Ginindza T.G., Hlongwana K.W. (2020). Health systems influence on the pathways of care for lung cancer in low- and middle-income countries: A scoping review. Glob. Health.

[7] Bertino J.R., Hait W., Golden L., Ausiello D. (2004). Principles of cancer therapy. Textbook of Medicine.

[8] Whittaker S., Marais R., Zhu A.X. (2010). The role of signaling pathways in the development and treatment of hepatocellular carcinoma. Oncogene.

[9] Agarwal E., Brattain M.G., Chowdhury S. (2013). Cell survival and metastasis regulation by Akt signaling in colorectal cancer. Cell Signal..

[10] Wang Z., Cole P.A. (2014). Catalytic mechanisms and regulation of protein kinases. Methods Enzymol..

[11] Drake J.M., Lee J.K., Witte O.N. (2014). Clinical targeting of mutated and wild-type protein tyrosine kinases in cancer. Mol. Cell Biol..

[12] Knosel T., Kampmann E., Kirchner T., Altendorf-Hofmann A. (2014). tyrosine kinases in soft tissue tumors. Pathologe.

[13] Winkler G.C., Barle E.L., Galati G., Kluwe W.M. (2014). Functional differentiation of cytotoxic cancer drugs and targeted cancer therapeutics. Regul. Toxicol. Pharmacol..

[14] Martin H.C., Grant W., John R.J., John D., Jogarao G., Atiqur R., Kimberly B., John L., Sung K.K., Rebecca W. (2002). Approval summary for imatinib mesylate capsules in the treatment of chronic myelogenous leukemia. Clin. Cancer Res..

[15] Druker B.J., Guilhot F., O’Brien S.G., Gathmann I., Kantarjian H., Gattermann N., Deininger M.W., Silver R.T., Goldman J.M., Stone R.M. (2006). Five-year follow-up of patients receiving imatinib for chronic myeloid leukemia. N. Engl. J. Med..

[16] Christopher F. (2007). Targeted chronic myeloid leukemia therapy: Seeking a cure. J. Mang. Care Pharm..

[17] Jiao Q., Bi L., Ren Y., Song S., Wang Q., Wang Y.S. (2018). Advances in studies of tyrosine kinase inhibitors and their acquired resistance. Mol. Cancer.

[18] Darwish I.A., Khalil N.Y., AlZeer M. (2020). ICH/FDA Guidelines-Compliant Validated Stability-Indicating HPLC-UV Method for the Determination of Axitinib in Bulk and Dosage Forms. Curr. Anal. Chem..

[19] Khandare B., Musle A.C., Arole S.S., Popalghat P.V. (2019). Analytical method development and validation of olmutinib bulk drug as per ICH Q2 guidelines by using RP-HPLC Method. J. Drug Deliv. Ther..

[20] Khalil N.Y., Darwish I.A., Alshammari M.F., Wani T.A. (2017). ICH Guidelines-compliant HPLC-UV Method for Pharmaceutical Quality Control and Therapeutic Drug Monitoring of the Multi-targeted Tyrosine Kinase Inhibitor Pazopanib. S. Afr. J. Chem..

[21] Latha S.T., Thangadurai S.A., Jambulingam M., Sereya K., Kamalakannan D., Anilkumar M. (2017). Development and validation of RP-HPLC method for the estimation of Erlotinib in pharmaceutical formulation. Arab. J. Chem..

[22] Ashok G., Mondal S., Ganapaty S., Bandla J. (2015). Development and validation of stability indicating method for the estimation of pazopanib hydrochloride in pharmaceutical dosage forms by RP-HPLC. Der Pharm. Lett..

[23] Bende G., Kollipara S., Kolachina V., Saha R. (2007). Development and validation of a stability indicating RP-LC method for determination of imatinib mesylate. Chromatographia.

[24] Hajmalek M., Goudarzi M., Ghaffari S., Attar H., Mazlaghan M.G. (2016). Development and validation of a HPTLC method for analysis of sunitinib malate. Braz. J. Pharm. Sci..

[25] Dutta D., Das S., Ghosn M. (2019). Validated HPTLC method for the determination of nintedanib in bulk drug. Proceedings.

[26] Vadera N., Subramanian G., Musmade P. (2007). Stability-indicating HPTLC determination of imatinib mesylate in bulk drug and pharmaceutical dosage form. J. Pharm. Biomed. Anal..

[27] Mhaske D.V., Dhaneshwar S.R. (2007). Stability indicating HPTLC and LC determination of dasatinib in pharmaceutical dosage form. Chromatographia.

[28] Reddy C.N., Prasad P., Sreedhar N.Y. (2011). Voltammetric behavior of gefitinib and its adsorptive stripping voltammetric determination in pharmaceutical formulations and urine samples. Int. J. Pharm. Pharm. Sci..

[29] Rajesh V., Jagathi V., Sindhuri K., Devala Rao G. (2011). Spectrofluorimetric method for the estimation of Erlotinib hydrochloride in pure and pharmaceutical formulations. E-J. Chem..

[30] Mandal B., Balabathula P., Mittal N., Wood G.C. (2012). Himanshu Bhattacharjee. Development and validation of a spectrofluorimetric method for the determination of Erlotinib in spiked human plasma. J. Fluoresc..

[31] Zawaneh A.H., Khalil N.N., Ibrahim S.A., Al-Dafiri W.N., Maher H.M. (2017). Micelle-enhanced direct spectrofluorimetric method for the determination of linifanib: Application to stability studies. Luminescence.

[32] Maher H.M., Alzoman N.Z., Shehata S.M. (2017). An eco-friendly direct spectrofluorimetric method for the determination of irreversible tyrosine kinase inhibitors, neratinib and pelitinib: Application to stability studies. Luminescence.

[33] Padmalatha H., Vidyasagar G. (2011). Development and validation of UV spectrophotometric method for the determination of erlotinib in tablet formulation. Imperial. J. Med. Org. Chem..

[34] Sankar F.G., Latha P.V., Krishna M.V. (2006). UV-spectrophotometric determination of imatinib mesylate. Asian J. Chem..

[35] Sankar D.G., Rajeswari A., Babu A.N., Krishna M.V. (2009). UV-spectrophotometric determination of dasatinib in pharmaceutical dosage forms. Asian J. Chem..

[36] Annapurna M.M., Venkatesh B., Chaitanya R.K. (2014). Analytical techniques for the determination of erlotinib HCl in pharmaceutical dosage forms by spectrophotometry. Chem. Sci. Trans..

[37] Khandare B., Dudhe P.B., Upasani S., Dhoke M. (2019). Spectrophotometric determination of vandetanib in bulk by area under curve and first order derivative methods. Int. J. PharmTech Res..

[38] Annapurna M.M., Venkatesh B., Chaitanya R.K. (2013). New derivative spectrophotometric methods for the determination of erlotinib hydrochloride (a tyrosine kinase inhibitor). Indo Am. J. Pharm. Res..

[39] Khandare B., Musle A.C., Arole S.S., Pravin V., Popalghat V. (2019). Spectrophotometric determination of olmutinib in bulk by area under curve and first order derivative methods and its validation as per ICH guidelines. J. Drug Deliv. Ther..

[40] Souria E., Amoon E., Ravarib N.S., Keyghobadia F., Tehrania M.B. (2020). Spectrophotometric methods for determination of sunitinib in pharmaceutical dosage forms based on ion-pair complex formation. Iran. J. Pharm. Res..

[41] Rani G.U., Chandrasekhar B., Devanna N. (2011). Extractive colorimetric method development and validation for erlotinib in bulk and tablet dosage form. J. Appl. Pharm. Sci..

[42] Balaram V.M., Rao J.V., Khan M.M., Sharma J.V., Anupama K. (2009). Visible spectrophotometric determination of imatinib mesylate in bulk drug and pharmaceutical formulations. Asian J. Chem..

[43] Abdel Karim S.E., Farghaly R.A., El-Nashar R.M., Abadi A.H. (2014). Spectrophotometric determination of imatinib mesylate using charge transfer complexs in pure form and pharmaceutical formulation. Chem. Rapid Commun..

[44] Syamittra B., Parasuraman S., Yeng W.Y., Ping Y.W., Thujithra J., Kumar J., Dhanaraj S.A. (2014). A review on adverse health effects of laboratory volatile solvents. Int. J. Clin. Ther. Diagn..

[45] Wennborg H., Bonde J.P., Stenbeck M., Olsen J. (2002). Adverse reproduction outcomes among employee in biomedical research laboratories. Scand. J. Work Environ. Health.

[46] Lindbohm M.L., Taskinen H.T., Sallman M., Hemminki K. (2007). Spontaneous abortions among women exposed to organic solvents. Am. J. Indust. Med..

[47] Wennborg H., Lennart B., Harri V., Gösta A. (2000). Pregnancy outcome of personnel in Swedish biomedical research laboratories. J. Occup. Environ. Med..

[48] Kristensen P., Hilt B., Svendsen K., Grimsrud T.K. (2008). Incidence of lymphohaematopoietic cancer at university laboratory: A cluster investigation. Eur. J. Epidemiol..

[49] Armenta S., Garrigues S., de la Guardia M. (2008). Green analytical chemistry. Trends Anal. Chem..

[50] Perry G.W. (2009). High Throughput Analysis in the Pharmaceutical Industry.

[51] Darwish I.A., Darwish H.W., Khalil N.Y., Sayed A.Y. (2021). Experimental and computational evaluation of chloranilic acid as an universal chromogenic reagent for the development of a novel 96-microwell spectrophotometric assay for tyrosine kinase inhibitors. Molecules.

[52] Görög S. (2018). Ultraviolet-Visible Spectrophotometry in Pharmaceutical Analysis.

[53] Ahmed S., Rasul A., Masood Z. (2011). Spectrophotometry in Pharmaceutical Analysis.

[54] Gore M.G. (2000). Spectrophotometry and Spectrofluorimetry: A Practical Approach.

[55] Mennen S.M., Alhambra C., Allen C.L., Barberis M., Berritt S., Brandt T.A., Campbell A.D., Castañón J., Cherney A.H., Christensen M. (2019). The evolution of high-throughput experimentation in pharmaceutical development and perspectives on the future. Org. Process Res. Dev..

[56] Welch C.J. (2019). High throughput analysis enables high throughput experimentation in pharmaceutical process research. React. Chem. Eng..

[57] Dunn P.J., Wells A.S., Williams M.T. (2010). Green Chemistry in the Pharmaceutical Industry.

[58] Agbenyega J. Green Chemistry in the Pharma Industry: Sustainable Pastures for Those Who Innovate. https://www.cas.org/resources/cas-insights/sustainability/green-chemistry-pharma-industry.

[59] Msingh R., Pramanik R., Hazra S. (2021). Role of green chemistry in pharmaceutical industry: A review. J. Univ. Shanghai Sci. Technol..

[60] Darwish I.A., Almehizia A.A., Sayed A.Y., Khalil N.Y., Alzoman N.Z., Darwish H.W. (2021). Synthesis, spectroscopic and computa-tional studies on hydrogen bonded charge transfer complex of duvelisib with chloranilic acid: Application to development of novel 96-microwell spectrophotometric assay. Spectrochim. Acta A.

[61] Darwish I.A., Khalil N.Y., Darwish H.W., Alzoman N.Z., Al-Hossaini A.M. (2022). Synthesis, spectroscopic and computational characterization of charge transfer complex of remdesivir with chloranilic acid: Application to development of novel 96-microwell spectrophotometric assay. J. Mol. Struct..

[62] Khalil N.Y., Al Qhatani M.N., Al Qubaisi K.A., Sayed A.Y., Darwish I.A. (2022). Development of two innovative 96-microwell-based spectrophotometric assays with high throughput for determination of fluoroquinolone antibiotics in their pharmaceutical formulations. J. Appl. Spectrosc..

[63] International Council for Harmonisation of Technical Requirements for Pharmaceuticals for Human Use (2022). ICH Harmonised Guideline, Validation of Analytical Procedure: Q2(R2).

[64] Keith L.H., Gron L.U., Young J.L. (2007). Green analytical methodologies. Chem. Rev..

[65] Gałuszka A., Konieczka P., Migaszewski Z.M., Namieśnik J. (2012). Analytical eco-scale for assessing the greenness of analytical procedures. Trends Anal. Chem..

[66] Płotka-Wasylka J. (2018). A new tool for the evaluation of the analytical procedure: Green Analytical Procedure Index. Talanta.

[67] Pena-Pereira F., Wojnowski W., Tobiszewski M. (2020). AGREE-analytical greenness metric approach and software. Anal. Chem..

